# A Retrospective, Observational Study on Antimicrobial Drug Use in Beef Fattening Operations in Northwestern Italy and Evaluation of Risk Factors Associated with Increased Antimicrobial Usage

**DOI:** 10.3390/ani11071925

**Published:** 2021-06-28

**Authors:** Isabella Nicola, Giovanni Gallina, Giulia Cagnotti, Paola Gianella, Flaminia Valentini, Antonio D’Angelo, Claudio Bellino

**Affiliations:** 1 Department of Clinical Science, Faculty of Veterinary Medicine, Université de Montréal, 3200 Rue Sicotte, St-Hyacinthe, QC J2S 7C6, Canada; isabella.nicola@umontreal.ca; 2 Department of Veterinary Sciences, Clinical Section, University of Turin, Largo Paolo Braccini 2, 10095 Grugliasco, TO, Italy; giovannigallina92@gmail.com (G.G.); giulia.cagnotti@unito.it (G.C.); paola.gianella@unito.it (P.G.); flaminia.valentini@unito.it (F.V.); antonio.dangelo@unito.it (A.D.)

**Keywords:** animal daily dose, antimicrobial usage, beef cattle

## Abstract

**Simple Summary:**

Antimicrobial usage in veterinary medicine is thought to be a source of antimicrobial resistance, with possible implications for human health. Certain antibiotics are considered critical for human health, and their use is being judiciously reduced in animal productions. The monitoring of antimicrobial consumption in animal production is key to lowering the risk of the development of antimicrobial resistance. With this study, we quantified antimicrobial usage in beef fattening operations in northwestern Italy before the implementation of a program intended to control antimicrobial usage in veterinary medicine. We found that antimicrobials defined as critical for human health (e.g., fluroquinolones) were often used also for metaphylactic treatment.

**Abstract:**

The abuse or misuse of antimicrobials in animal production is thought to be a potential factor in the development of antimicrobial resistance in veterinary and human medicine. With this study, we wanted to quantify antimicrobial usage in beef fattening operations in northwestern Italy and to identify factors potentially influencing antimicrobial usage. The sample was composed of 26 beef fattening operations that import heifers and bulls from France. Data were extracted from the 2014 and 2015 treatment registers kept by the farmers. The mean (±SD) number of animal daily doses per animal (nADDa) per year for each farm was 3 (±2.1) during the study period (2014–2015). Group antimicrobial treatments (57.5% of all treatments) were often administered orally (70.5%) and consisted overwhelmingly of doxycycline (97%). Individual treatments (42.5% of all treatments) were administered parenterally (98.1%) and the most often used active substances were florfenicol (19.9%), marbofloxacin (19.5%), and tylosin (12.4%). There was a negative correlation between the nADDa for total and group treatments and average batch weight at arrival and between the amount of straw added per animal per day and the nADDa (*p* ≤ 0.05). Our data show that antimicrobials critical for human medicine were often used in beef fattening operations in northwestern Italy before the European guidelines for the prudent use of antimicrobials in veterinary medicine were issued. Additionally, the use of antimicrobials as a preventive group treatment was still widespread, mostly in lighter weight animals.

## 1. Introduction

Antimicrobial resistance is a growing concern in both human and veterinary medicine. According to a 2014 review, an estimated 700,000 human deaths per year may be attributed to antimicrobial resistance, with 10 million deaths per year estimated by 2050 if antimicrobial consumption is not controlled [1]. Antimicrobial abuse and misuse in human medicine are considered the leading causes of the development of antimicrobial resistance in human pathogens; however, livestock and environment may also be a potential source of resistance [2]. The development of antimicrobial resistance in zoonotic pathogens affecting human patients, such as *Campylobacter* spp., enterococci, *Escherichia coli*, *Salmonella* spp. and *Staphylococcus aureus*, is increasing [3]. Though the mechanism of antimicrobial resistance transmission from livestock to human has not yet been fully elucidated, such a transmission is likely to happen, generally via environmental contamination of the food chain [2,3].

Furthermore, national monitoring programs in Europe have reported an increase in antimicrobial resistance in human pathogens, possibly correlated with the usage of certain active substances in food-producing animals [4]. In an effort to better control antimicrobial usage in veterinary medicine in Europe, the European Commission issued specific guidelines in 2015 that recommended the prescription of antimicrobial drugs based on a susceptibility test, the use of metaphylaxis only when needed, and the discontinuation of all types of prophylactic treatment [5]. Furthermore, guidelines included limitations in veterinary medicine of the use of antimicrobials considered critical for human medicine, listed as highest priority critically important antimicrobials (HPCIA) by the World Health Organization (WHO) [6]. When the new European Regulation on veterinary medical production goes into force in early 2022, the use of antimicrobial drugs for prophylactic and metaphylactic use will be restricted by law, as will the use of HPCIA in veterinary medicine [7].

Monitoring antimicrobial consumption is the first step to reduce their usage and to identify situations where antimicrobial administration could be potentially avoided. Mandatory or voluntary plans for the reduction in antimicrobial usage, particularly the category comprising HPCIA (e.g., fluoroquinolones and third and fourth generation cephalosporines), have been implemented (e.g., the Netherlands, Denmark, Finland, Sweden, United Kingdom, France, and Belgium), and guidelines for the responsible use of antimicrobials have been distributed [8]. The plans included national monitoring of antimicrobial sales and consumption in various production categories [9,10]. The countries where the plans have been implemented have reported a considerable reduction in the sale of certain antimicrobials and in antimicrobial resistance [8].

A national plan for monitoring antimicrobial resistance and usage was issued in Italy in 2017 [11], and guidelines for the correct use of antimicrobials in 2018 [12]. 

Current Italian legislation regulating veterinary drugs does not prohibit the use of antimicrobial drugs for metaphylactic or prophylactic treatment in livestock, whereas the use of antimicrobials as growth promoters is prohibited, as stated by the European ban on growth promoters [13]. Nonetheless, the use of antimicrobials for prophylactic or metaphylactic treatment is not recommended and may only be done following the recommendation of a veterinarian. 

Under the provisions of Italian Decree Law 193/2006, veterinary drugs can be sold only by pharmacists on prescription by a veterinary practitioner. Cattle farms can be authorized by the local veterinary service to hold drug stocks, but they must keep accurate records of the drugs they purchase and use. The veterinary practitioner was responsible for recording in the paper register, once the drugs are purchased, the following information for each product in stock: date of purchase/use, package size and number of packages, amount of drugs administered, treatment length, and identification number of the animal(s) that received the drug(s). Veterinary practitioners can prepare a treatment plan for the most common diseases, which the farmer then can consult as needed. In farms not keeping a drug stock, veterinary prescriptions should include the identification number of the animals treated with the prescribed drug. Irrespective of whether the farmers are permitted to keep a drug stock or not, they must also maintain records of treatment administered to the animals. A paper register is then completed with information on the drug supplier’s name and address, the package identification number, the amount of drug administered in 24 h, the animal identification number, the date therapy initiated and completed, and the trade name of the drug. All paper registers and veterinary prescription invoices must be kept for 5 years; the registers are checked at least once a year by an official veterinarian. In Italy, since the beginning of 2019, the prescription of drugs and the management of farm drugs stocks has taken place electronically.

Italy is one of Europe’s leading meat producers, accounting for around 11% of meat produced annually in the European Union [14,15,16]. The beef cattle sector mostly comprises intensive fattening operations (70–75%) primarily located in the north of Italy, which import cattle mainly (80%) from France [15]. While the northeast ranks first in meat production in Italy, the northwest is the second largest meat producer, accounting for about 40% of meat production [17]. In 2014 and 2015, Italy imported 1,864,161 (956,398 and 907,763) beef cattle, 60% of which were imported to the northeast and 35% to the northwest (source, National Livestock Register). Hence, beef fattening operations may be an important contributor in antimicrobial usage in Italian livestock. The breeding systems of imported youngstock are similar for fattening units in the northeast and the northwest, with indoor loose housing of the animals in multiple pens. According to data from an analysis published in 2007, the units are larger in the northeast [15].

Recent studies investigated the monitoring of antimicrobial use in the cattle fattening sector in the northeast [18,19]. To our knowledge, data from the northwest are lacking, despite its important role in meat production. The aim of the present study was to quantify antimicrobial usage in fattening operations in the northwest of Italy before the national plan for antimicrobial consumption and resistance control (2017) went into effect in order to have data for future comparisons. A secondary aim was to identify factors potentially influencing antimicrobial usage. 

## 2. Materials and Methods

### 2.1. Farm Recruitment

The present study was performed in collaboration with the *Associazione Servizi Agricoli Zootecnici* (ASAZ), an agency accredited by the regional government. It provides consultancy on animal health and herd management as part of the Rural Development Program for Piedmont 2014/2020. The Association is composed of six food animal veterinarians who work with cattle farmers and provide their services to farmers operating beef cattle fattening or veal calf units in Piedmont. 

Thirty fattening operations that import beef heifers and bulls at age 8–12 months from France agreed to participate in the study. Each farm was visited once during 2016. During the visit, the farmer was asked to complete a questionnaire investigating management, structure, and animal characteristics. Data were also obtained from the farm’s paper registers about treatments and animal movement. 

### 2.2. Animals and Farm Data 

The questionnaire included items on: average animal weight at the beginning and the end of the fattening period, main breed imported, medical history/treatment/vaccinations before arrival, arrival procedures (vaccinations, parasiticide administration, antimicrobial prophylactic/metaphylactic treatment, quarantine period, fattening group formation), animal health management (daily check, frequency of veterinary visits, confinement of sick animals), and housing characteristics (kind of ventilation, space in m^2^/animal, animals/pen, frequency of straw adding, cleaning and disinfection frequency, depopulation period). At the beginning and at the end of the production cycle, each batch entering or exiting the farm was weighed and the total weight was divided by the number of the animals in the batch. The average weight at the beginning and at the end of the production cycle was estimated by the farmer based on data collected during the two years of the study. Furthermore, mortality data were obtained by an official record that farmers must keep, which includes animals entering and leaving the farm, as well as deaths. The records for each year of the study were consulted to obtain the number of deaths and the number of animals at risk. Annual mortality was calculated for each year by dividing the number of deaths by animals at risk. Animals at risk of dying were defined with the same method used to define animals at risk of being treated, as explained in the following subsection (“Antimicrobial consumption data”). For each farm, we obtained an average mortality rate for two consecutive years. Table 1 and Table 2 present the characteristics of animals and farms. 

### 2.3. Antimicrobial Consumption Data 

Data on the amount of antimicrobials used and treatments for 2014 and 2015 were extracted from the registers or the prescription drug invoices kept by the farmers, including: antimicrobial trade name, pharmaceutical form (oral or parenteral), package size (mg for powder and mL for liquid), and total number of packages consumed. 

Treatment data were extracted from the registers. Based on the data from the two types of records, we were able to classify antimicrobial drug treatments as: oral vs. parenteral and individual vs. group. Treatments were defined as “group” when they were identified in the records by batch number and “individual” when they were identified in the records by animal identification number. The active substance was identified from its trade name listed in the national drug register of the Italian Ministry of Health.

Antimicrobial drug usage was quantified based on the animal daily dose (ADD) methodology described in Jensen et al., 2004 [9,20]. The ADD is defined as the average maintenance antimicrobial dose of a drug for the main indication in a specified animal species [9]. The ADD is based on the recommended dose approved by the pharmaceutical companies marketing the drug in Italy. For the ADD, the antimicrobial was identified according to the Anatomical Therapeutic Chemical classification system for veterinary medicinal products (ATCvet) [21]. When an antimicrobial was indicated for more than one disease (e.g., respiratory disease and mastitis), the dose was that indicated for the more frequent disease in fattening operations (respiratory, gastrointestinal, foot diseases). 

When the active substance had multiple dosages in different commercial products or when a range of dosages was suggested by the same pharmaceutical company, the mean was used as the ADD. International units were converted according to the converting factors published by the European Medicines Agency (EMA) for each active substance (spiramycin 3200 IU/mg; procaine benzylpenicillin 1667 IU/mg, colistin sulphate 20,500 IU/mg) [22]. The ADD for long-acting drugs was recalculated from the recommended dosage to obtain a 24 h dose by dividing by the long-acting factor (LA factor), defined as the number of days in treatment after one application of the drug. Long-acting factors were obtained from the literature and the summary of product characteristics [20,23,24].

The number of ADD (nADD) was calculated with the following equation:

(1) $$nADD=\frac{Total\;amount\;of\;drug\;administered\;\left(mg\right)}{ADD\;\left(\frac{mg}{kg}\right)\times average\;animal\;weight\;\left(kg\right)}$$

The total amount of drug administered was calculated from the drug records and prescription invoices. The average animal weight was the average weight between the beginning and the end of the production cycle recorded for each farm. 

For each farm, the nADD administered to each animal (nADDa) per year in the 2 years of the study was an average of the nADDa calculated for each year. The nADDa was obtained by dividing the nADD by the total number of animals at risk of having been treated on each farm in each year (2014 and 2015), as shown below:

(2) $$nADDa=\frac{nADD}{total\;number\;of\;animal\;at\;risk}$$

Since the farms did not practice the “all-in all-out” system, the total number of animals at risk of treatment was defined as the number of animals reported on the official record of each farm at the beginning (1 January) of each year (2014 and 2015) in addition to the animals that entered the farm over the following 12 months. Animals sold or slaughtered in 2014 were potentially at risk of treatment during the year 2014 but not counted in the subsequent year (2015).

The antimicrobial usage records were reviewed to obtain the used daily dose (UDD) for each drug, i.e., the dose actually administered to an animal. The UDD of each drug was calculated with the following equation:

(3) $$UDD=\frac{total\;amount\;of\;drug\;administration\;\left(mg\right)}{average\;animal\;weight\;\left(kg\right)\times number\;of\;applications\;}$$

The number of applications comprises all treatments given to an animal and was obtained by multiplying the number of animals treated by treatment days (Equation (4)).

(4) number of applications=number of animals treated×number of days of treatment

The UDD/recommended dose (24 h) ratio was calculated to determine compliance with dosing. A ratio between 0.8 and 1.2 was considered appropriate. A ratio <0.8 or >1.2 was defined as under- or overdosing, respectively [25]. 

### 2.4. Statistical Analysis

Statistical analysis was performed using the R freeware statistical software package v.3.4.1. Categorical variables are reported as frequency, percentage, or both; numerical variables were reported as median (min, max). Since differences in the weight of animals housed together have been considered as a possible risk factor for antimicrobial usage [24], we divided the farms into three groups based on the maximal weight difference between batches’ average weight at arrival for each farm: <50 kg, 50–100 kg, >100 kg. The nADDa is reported as the mean ± standard deviation (SD) (median, max); the UDD/recommended dose ratio is reported as the mean ± SD (min and max) and the mean ± SD of percentage of under-, normal, or overdosing for each farm. In order to identify possible risk factors for antimicrobial usage, Spearman’s correlation coefficient was used to determine the correlation between the nADDa, both total and divided by type of treatments (individual and group), and numerical variables (Table 1). The correlation between individual and group treatments was also evaluated. The Wilcoxon rank-sum test was performed to determine differences in nADDa based on categorical variables (Table 2). For variables involving more than two categories, pair-wise comparisons were made using the Wilcoxon rank-sum test and the *p* value was corrected with the Bonferroni method. When the farm distribution within the different categories was skewed (e.g., parasiticides at arrival 23 Yes vs. 3 No), the test was not performed. Variables that significantly influenced the nADDa were then tested against each other to determine possible associations. *p* was set at 0.05.

## 3. Results

Four of the 30 farms that had agreed to participate in the study provided incomplete information for antimicrobial prescriptions and were excluded from the final analysis. The sample was 26 farms that engaged the services of five veterinarians. 

### 3.1. Animal and Farm Data

The average total number of animals at risk per year was 32,360. 

The farms generally imported French beef breeds, including Blonde d’Aquitaine, Limousine, Charolais, Aubrac and French cross breed cattle. An accurate estimation of the percentage of breeds imported by each farm was not available. The median average weight at the end of the fattening cycle was 665 kg (475, 750). All farms had a vaccination protocol against respiratory viruses (bovine herpesvirus type 1, bovine parainfluenza-3 virus, bovine respiratory syncytial virus and bovine viral diarrhea virus) in place and 88.5% (23/26) also vaccinated against respiratory bacteria (23/23 against *Mannheimia haemolytica*; 2/23 against *Histophilus somni*). The median number of days between arrival and vaccination was 1 (0, 7). All farms had a planned quarantine period. The animals were checked at least twice a day on all 26 farms. Veterinary visits were regularly scheduled on 23 farms and 23 (88.5%) farmers practiced depopulation in quarantine pens between batches. All farms had separation fences that allowed nose-to-nose contact and all farms had a straw bedding system (Table 1 and Table 2).

### 3.2. Antimicrobial Consumption 

Overall, 821.7 kg of antimicrobials were used by the 26 farms during 2014 and 2015; 57.2% were orally administered and were mostly tetracyclines (91.4%). The amount (kg) of parenterally administered antimicrobials was lower (42.8%), but there was more variety of active substances than the orally administered. The antimicrobials most often used in parenteral administration were fenicoles (38.7%) and macrolides (23.8%) (Table 3).

Overall, the mean (±SD, median, max) nADDa for each farm was 3 (±2.1, 2.6, 8.3). The mean (±SD, median, max) nADDa for group treatments was 1.7 (±1.9, 1.2, 6.8); antimicrobial treatments were orally administered in 70.5% and parenterally administered in 29.5% of cattle. Oral formulations were composed primarily of doxycycline (97%). Parenteral formulations were composed mainly of long-acting macrolides, such as tulathromycin (41.5%) and tildipirosin (26.8%), and formulations containing florfenicol (6.8%), alone or in combination with flunixin meglumide. Mean (±SD, median, max) nADDa in individual treatments was 1.3 (±0.7, 1.4, 3), 98.1% of which was administered parenterally. The most frequent active substances were florfenicol (19.9%), marbofloxacin (19.5%), and tylosin (12.4%) (Table 4).

For all of the treatments, the mean UDD/recommended dose ratio was 0.9 (±0.26; 0.56, 1.62). The average UDD/recommended dose ratio for group treatments was 0.77 (±0.29; 0.24, 1.23). On average, 23.5% (±35.9%) of the orally administered group treatments were underdosed, 41.2% (±44.1%) were administered at the correct dosage, and 35.3% (±49.3%) were overdosed, while 75.5% (±34.3%) of the parenterally administered group treatments were underdosed, 17.9% (±30.4%) were administered at the correct dosage, and 6.5% (±14.6%) were overdosed. The mean UDD/recommended dose ratio for individual treatments was 0.98 (±0.25; 0.58, 1.62). Individual orally administered treatments were underdosed in 14.3% (±37.8%) of cases, correctly administered in 28.57% of cases (±48.8%), and overdosed in 57.1% of cases (±53.5%), while parenterally administered individual treatments were underdosed in 48% of cases (±21.9%), correctly administered in 24.7% of cases (±13.3%), and overdosed in 27.4% of cases (±17%). Further details on the UDD/recommended dose ratio are reported in Table 5.

### 3.3. Associations between Antimicrobial Consumption and Possible Risk Factors

No associations were found between mortality and nADDa considered as a whole and by type of treatment (individual or group) (*p* > 0.05). No association was found between group and individual nADDa (*p* > 0.05). A negative correlation was found between nADDa and average batch weight at arrival for total (*p* < 0.05, r = −0.47) and group treatments (*p* < 0.05, r = −0.56). A negative correlation was also found between the amount (kg) of straw added per day per animal and the nADDa by group (*p* < 0.05, r = −0.44) and total treatments (*p* = 0.05; r = −0.38).

Group and total treatment nADDa statistically differed between farms that performed antimicrobial prophylactic/metaphylactic treatment regularly at arrival and farms that did not. No difference was observed for individual treatments. 

Farms that regularly practiced antimicrobial prophylactic/metaphylactic treatment had a higher nADDa (*p* < 0.05) for group (2.6 ± 2; 2, 6.8) and total treatments (3.8 ± 2.3; 3.3, 8.3) compared to farms that did not practice antimicrobial prophylactic/metaphylactic treatment at arrival (group 0.5 ± 0.6; 0.1, 1.8; total 1.8 ± 1.1; 1.5, 4.4). The median average batch weight at arrival was lower (*p* < 0.05) for the farms that regularly practiced antimicrobial prophylactic/metaphylactic treatment (310 kg; 195, 450) compared to the other farms (400 kg; 280, 475). No other differences were noted for the other categories. Table 2 presents the nADDa for the total, individual and group treatments for each parameter and category.

## 4. Discussion

Antimicrobial usage monitoring in cattle has attracted growing interest due to the increase in antimicrobial resistance and the potential impact of livestock production on its development [4]. Using the nADDa calculation method allows us to determine the amount of antimicrobial use and to compare it between animal species and active compounds [9]. The amount (in kg) of active compound used gives only a raw idea of antimicrobial usage because it is influenced by animal weight and active compound potency [9]. In other words, if only the amount (in kg) of antimicrobial used is calculated, then the usage of a more-potent antimicrobial would be underestimated, as would the usage in lighter weight animal species such as poultry. For example, in the present study, the amount (in kg) of fenicoles used was higher than that of fluoroquinolones and macrolides; however, the difference appears less remarkable when we observe the nADDa in these categories. 

Nevertheless, one of the limitations of this method of calculation is that it requires the weight of the animals at the time of antimicrobial administration [10]. As the exact animal weight at time of treatment is hard to determine, average weights for beef cattle categories were suggested: 300, 500, and 600 kg [9,10,19,27]. 

However, because of the wide range of weights in the present sample and the fact that none of the published values fitted well with all farms, we calculated a mean weight between the beginning and the end of the fattening period. This solution is shared by previous studies [18,28]. While it is probably a better fit for our population than the published weight classes, this estimation still posed limitations owing to the large interval between weights at the time of treatment [29]. 

Another limitation of this retrospective study is the estimation of animals at risk in our calculations, since we were unable to individually follow each batch that entered the farms during the two years of the study period. The length of fattening cycle could have varied widely by farm and by weight of animals at arrival. Considering that some farms had a wide range of arrival average weights, a calculation of the number of animals at risk based on an average length of the fattening cycle was difficult to achieve. However, this estimation could have led to underestimation of nADDa used. 

The average nADDa per year per farm during the 2-year study period was 3. The nADDa reported for poultry, pigs, and veal calves is usually higher than that reported for adult cattle. A German study based on data collected between 2006 and 2007 reported a higher nADDa for piglets (60.86), fattening pigs (28.6) and calves (8.33) and a lower nADDa for dairy (2.75) and beef cattle (0.08) [30]. In a Dutch study, the animal categories with the highest nADDa were veal calves (20.88 in 2016), pigs (8.87 in 2016), broilers (10.19 in 2016), turkeys (26.42 in 2016), and rabbits (40.93 in 2016) [28]. The reported nADDa in beef cattle was lower compared to our data (1.07 in 2016, 1.15 in 2014, and 1 in 2015) [28]. However, since neither the German nor the Dutch studies divided the beef cattle by production categories (cow-calf or fattening units), the presence of other production categories (e.g., cow-calf operations) may have mitigated the nADD for beef cattle. Furthermore, a 2007 study involving 20 conventional farms in the United States (Wisconsin) reported the use of 5.43 nADD/cow per year [31]. Similar results were reported for the Netherlands, where a 7-year study involving 94 dairy farms reported an average nADDa of 5.86 [32]. A more recent study reported a lower nADDa for dairy herds (three in 2016) in the Netherlands [28]. 

Furthermore, the result of the present study was similar to that reported by Diana et al. for fattening operation units in northeastern Italy [19].

The oral route was principally used for group treatments and the nADDa in oral administration accounted for 41.2% of total treatments. It is not surprising that this percentage was lower compared to previous studies on veal calves, in which the majority of treatments were group treatments and administered orally [20,24,33]. However, Merle et al. reported higher oral antimicrobial usage not only in calves but also in beef cattle [34]. Furthermore, oral administration (amount in kg) was preferred over parenteral administration in Canadian feedlot studies [35,36]. 

In our sample, the active compound most often used in oral treatments was doxycycline. Tetracyclines were the most frequently used oral antimicrobials in both veal calves and beef fattening cattle according to studies conducted in Belgium, Denmark and Western Canada [20,36,37,38]. Oral tetracyclines have been used as prophylactic/metaphylactic treatment for the control of bovine respiratory disease (BRD) in post-weaned beef cattle at arrival in the feedlots [39]. At the time of writing, doxycycline was used off-label in fattening operations in Italy, as registered for calves. However, youngstock have a functionally developed rumen and there is a paucity of studies describing the pharmacokinetics of these molecules in functional ruminants. We believe that this practice should be discouraged as it potentially contributes to the development of antimicrobial resistance, mostly in the case of underdosing [36]. 

In group treatments by parenteral administration, the active substances most often used in the present study were tulathromycin and tildipirosin, two long-acting macrolides indicated for individual and metaphylactic treatment of BRD. They are widely used in BRD prevention and treatment thanks to their pharmacokinetics, which includes extended distribution in pulmonary tissue and slow elimination [40,41]. Tulathromycin was largely used for group treatments in veal calves and beef cattle, as reported in Swiss and Western Canadian studies [24,36]. Recent studies involving fattening operations in northeastern Italy reported a large use of macrolides, though the percentage by which they were administered in group or individual treatments was not reported [18,19]. 

Oral formulations for individual treatment were seldomly used in the present study, as has been reported for Swiss veal calves [24]. Individual treatments were mainly administered parenterally; the three active substances most often used were florfenicol, marbofloxacin, and tylosin. In Italy, florfenicol is indicated for the treatment of respiratory disease in cattle, marbofloxacin for respiratory disease and mastitis, while tylosin has multiple indications, including respiratory disease, mastitis, metritis, and foot rot [42,43,44]. Florfenicol was also one of the most commonly used antimicrobials for individual parenteral treatments in veal calves according to a Belgium study [20]. Moreover, it was largely used in fattening operations in northeastern Italy and on feedlots in Canada [18,35,36]. Fluoroquinolones were among the antimicrobials most frequently used in Swiss veal calves, while tylosin was largely used in group treatments in Swiss and Belgian veal calves [20,24].

Our findings show that group and individual parenteral treatments were often underdosed. Weight estimation may not have been accurate, possibly influencing the dosage. However, several UDD/recommended dose ratios were too low, even in lower weight animals. A similar tendency to underdose, depending on the active substance and treatment type, was reported in previous studies on monitoring of antimicrobial usage in cattle and pigs [18,20,25,34].

This could be correlated with the underestimation of body weight at treatment [20]. Furthermore, antimicrobial underdosing can lead to antimicrobial resistance, resulting in a greater threat to animal and human health [38,45]. Group antimicrobial administration, often oral, in feedlot and veal calves has been correlated with increased bacterial resistance in feces [38,46,47]. In addition, active substances such as oxytetracycline seem to be more often associated with antimicrobial resistance and co-resistance [48]. Many BRD pathogens are resistant to tetracyclines and macrolides, both of which were widely used in the present sample, and their abuse has been correlated with antibiotic resistance by human pathogens [4,48,49]. Furthermore, fluoroquinolones in food-producing animals have been associated with resistance in human pathogens [4]. Fluoroquinolones and macrolides, which were also found to be largely used in the present sample, are listed as HPCIA according to the WHO [6].

The European Commission guidelines were first issued during the second year of the study and they constitute a first step toward reducing the use of HPCIA in veterinary medicine [5]. This implies that for most of the period of the present study (2014–2015), people who work in the beef sector were probably not fully aware of the importance of these antimicrobials in human medicine. Comparison of our data with more recent studies indicates that HPCIA are still largely used in beef cattle operations in Italy [19]. However, it is worth noting that these data were collected in a period in which guidelines on the prudent use of antibiotics had already been published, but in Italy, there were still no restrictions regarding the use of HCPIA in livestock. When the new European Regulation of 2019/6 on veterinary drugs goes into effect in early 2022 in all EU Member States, we may expect harmonization of the control of antimicrobial usage. For example, a national monitoring plan will be mandatory for antimicrobial usage based on a comparable unit of measure across the EU. In addition, antimicrobial usage for prophylaxy will be strictly limited to individual animals or small groups considered at high risk, and the use of antimicrobials considered important for human medicine may be completely prohibited or permitted if they are the only molecule possible for used [7].

Animal health and welfare management need to be improved to reduce antimicrobial use. The misuse of antimicrobials should also be reduced. Veterinarians can have an important impact in farmers’ treatment choice and they can play a key role in educating farmers and raising awareness of antimicrobial resistance and ways to prevent it [50]. In Canada, for example, antimicrobial use has been reduced through new management protocols that limit the administration of oral tetracyclines; this reduction was accompanied by a slight increase in individual treatment [36]. Identifying critical management points correlated with elevated antimicrobial usage may be as important as monitoring antimicrobial use itself.

We found few associations between nADDa and farm management and structural factors. The fact that regular application of antimicrobial prophylactic/metaphylactic treatment at arrival influenced the nADDa for group treatments is easy to understand, but it is also worth noting that it influenced the total nADDa as well. Additionally, both total and group nADDa were correlated with average batch weight at arrival. Antimicrobial prophylactic/metaphylactic treatment at arrival was more often administered to lighter weight calves, which are known to be at greater risk for developing BRD, which is the most common disease in feedlot cattle [51]. The use of antimicrobials as a preventive measure in lighter weight calves could have been a management decision to protect these animals at higher risk of disease. However, BRD was also implicated in growth reduction rates in animals, mostly when treated more than once [51,52,53]. The greater antimicrobials use for prophylaxis/metaphylaxis in lighter weight animals may also have been a management choice when purchasing cheaper animals. Nevertheless, since animal weight in our study refers to an average for the year, we believe it is unlikely that the lower average weight of the batches was always due to the presence of unhealthy animals. However, individual nADDa did not change with antimicrobial prophylactic/metaphylactic treatment at arrival, nor was there a correlation with group nADDa. In addition, mortality was not correlated with total, group or individual nADDa. Our data show that antimicrobial prophylactic/metaphylactic group treatment at arrival did not seem to reduce further individual antimicrobial treatments or influence herd mortality. Group treatments were generally administered to animals at higher risk of developing BRD, which could explain why no differences were found.

Less use of straw bedding per animal was correlated with higher antimicrobial use in group and total treatments. The amount of added straw per animal per day influenced pen humidity and hygiene and mitigated cold stress during winter [54,55]. Greater amounts of straw are known to reduce the emission of ammonia [56], which can impair respiratory defense, predisposing mice and swine to rhinitis; similar effects on cattle have been suggested [57,58]. While less straw may in some way contribute to the development of BRD, another explanation is that farms where less straw per animal per day was used could have had a higher incidence of BRD and would have administered more group treatments for BRD control.

Few correlations between farm management and structure and BRD were found; a plausible explanation is that, slight differences aside, the farms in the present sample were similar. They were followed by veterinarian practitioners who collaborated and probably adopted a common line in recommendations to the farmers.

Another characteristic identified by previous studies was the correlation between antimicrobial usage and breed. Greater antimicrobial usage has been reported for Limousine and Blonde d’Aquitaine compared to other breeds and mixed cross breeds [59]. None of the farms in the present sample imported a single breed and the data on the distribution of breeds imported within and among the farms were not accurate enough to obtain valid results with statistical analysis. Diana et al. also reported a lighter weight for Limousine and Blonde d’Aquitaine at the beginning of the production cycle, which could partly explain why the antimicrobial usage for these two breeds was higher compared to others [59]. Though we did not evaluate antimicrobial usage in relation to breed, we also noted a correlation between animal weight and antimicrobial usage [59].

The use of treatment records to monitor antimicrobial use could be a limitation of the present study, since records are known to underestimate antimicrobial use and some records could be missing [31,60]. According to Italian law, treatment records must be signed by veterinary practitioners working with the farmers and checked once a year by public health veterinarians. Prescriptions and drug stock records must also be kept. We cross-referenced prescriptions and antimicrobial stock records to double check treatment records. Finally, the fact that many antimicrobials were underdosed strengthens the point that the use of treatment records is a minor limitation of the present study.

## 5. Conclusions

Our findings show that beef cattle fattening operations in 2014–2015 appeared to use fewer antimicrobials than in veal calves, pigs, and broilers. The main active substances are all indicated for BRD treatment, underlying the importance of this disease in this type of animal production. Furthermore, our study shows that HPCIA were largely used in beef cattle operations before the implementation of European Guidelines and Regulation, and offers a base for comparison in future studies to evaluate guidelines and regulation effectiveness in reducing antimicrobials usage in livestock. Another finding is the tendency to underdose antimicrobials, particularly when administered orally. This should be interpreted with caution, since accurate information about animal weight at treatment was unavailable. Further studies involving a greater range of farm types are needed to identify other potential factors correlated with antimicrobial usage.

## Figures and Tables

**Table 1:** Characteristics of the farms and the animals of 26 beef calves fattening operations located in northwestern Italy are reported as the median, minimum, and maximum for the two-year period (2014 and 2015).

Parameter	Median	Min	Max
Average number of animals/year	886	151	4200
Mortality/year (%)	0.9	0.14	4.94
Average weight at the beginning of production cycle (kg)	346	195	475
Average weight at the end of the production cycle (kg)	665	475	750
Interval between veterinary visits (days)	7	1	15
Space allowance in quarantine pens (m^2^/animal)	4.6	3.4	37.4
Space allowance in fattening pens (m^2^/animal)	4.7	3.4	6.7
Space allowance in hospital pens (m^2^/animal)	9.2	2.9	30
Number of animals/pen in quarantine area	14	6	60
Number of animals/pen in fattening area	9	5	20
Number of animals/pen in hospital pens area	3	1	9
Frequency of straw adding (days)	2	1	17
Amount of straw added per animal per day (kg)	3.1	1	5.7
Cleaning frequency in quarantine area (days) ^a^	20	10.5	91
Cleaning frequency in fattening area (days) ^b^	20.5	12.5	53
Cleaning frequency in hospital pens area (days) ^c^	20.5	10	60
Disinfection frequency in quarantine area (days) ^d^	20.5	15	365
Disinfection frequency in fattening area (days) ^e^	53	12.5	365
Disinfection frequency in hospital pens area (days) ^f^	25	1	365
Duration of depopulation period in quarantine area (days)	7	0	30

^a^ Including the 2 farms that had a scraper in the quarantine area. ^b,c^ Calculated only for the farms without a scraper in the fattening (*n* = 14) and the hospital pens (*n* = 20) area. ^d,e,f^ Calculated only for those farms that practiced disinfection in the quarantine (*n* = 24), the fattening (*n* = 21), and the hospital pens (*n* = 24) area.

**Table 2:** Total, individual and group treatment number of animal daily doses per animal (nADDa) are reported as mean ± standard deviation (sd) (median, max) for each parameter of the categorical variables analyzed in 26 fattening operations located in northwestern Italy. Most characteristics are reported as present (“Yes”) or absent (“No”); except for “Maximal weight difference between average batch weight at arrival (kg)”, “Bedding removal in fattening area” and “Bedding removal in fattening area”, which were divided in 3 groups.

Parameters	Category	n° Farms	Percent	Mean of nADDa ± SD (Median, Max)		
				Total Treatments	Single Treatments	Group Treatments
Purchase at least the 10% of females	Yes	10	38.5%	3.2 ± 2.1 (3.2, 8.3)	1.2 ± 0.8 (1.2, 2.6)	2.1 ± 2 (1.6, 6.8)
	No	16	61.5%	2.9 ± 2.1 (2.5, 8.2)	1.4 ± 0.7 (1.5, 3)	1.5 ± 1.8 (0.8, 6)
Maximal weight difference between batches’ average weight at arrival (kg)	<50	7	26.9%	3.1 ± 2.6 (2.6, 8.3)	1.3 ± 1 (1.6, 3)	1.8 ± 2.4 (1, 6.8)
	50–100	13	50%	3.2 ± 2.3 (2.6, 8.2)	1.3 ± 0.7 (1.4, 2.6)	1.9 ± 2 (1.2, 6)
	>100	6	23.1%	2.4 ± 1.2 (2.5, 3.8)	1.2 ± 0.5 (1.2, 1.7)	1.2 ± 1 (1.1, 2.5)
Pre-arrival health information	Yes	1	3.8%	7	1.6	5.4
	No	25	96.2%	2.8 ± 2 (2.6, 8.3)	1.3 ± 0.7 (1.4, 3)	1.6 ± 1.8 (1, 6.8)
Thorough physical examination at arrival	Yes	8	30.8%	2.5 ± 1.2 (2.5, 4.4)	0.9 ± 0.4 (0.9, 1.6)	1.6 ± 1.2 (1.5, 3.9)
	No	18	69.2%	3.2 ± 2.4 (2.8, 8.3)	1.4 ± 0.7 (1.5, 3)	1.8 ± 2.1 (0.9, 6.8)
Vaccination against bacteria	Yes	23	88.5%	3 ± 2.2 (2.6, 8.3)	1.3 ± 0.7 (1.4, 3)	1.8 ± 2 (1, 6.8)
	No	3	11.5%	2.7 ± 1.2 (2.3, 4)	1.2 ± 0.7 (1.6, 1.6)	1.5 ± 0.9 (1.2, 2.4)
More than 1 days between arrival and vaccination	Yes	10	38.5%	2.8 ± 2.3 (2.1, 8.3)	1.1 ± 0.8 (0.9, 3)	1.7 ± 2.2 (1.1, 6.8)
	No	16	61.5%	3.1 ± 2.1 (2.9, 8.2)	1.4 ± 0.6 (1.5, 2.6)	1.7 ± 1.7 (1.1, 6)
Parasiticides at arrival	Yes	23	88.5%	2.7 ± 2 (2.3, 8.3)	1.2 ± 0.7 (1.1, 3)	1.5 ± 1.8 (0.8, 6.8)
	No	3	11.5%	5.1 ± 2.8 (4, 8.2)	1.9 ± 0.3 (1.7, 2.2)	3.3 ± 2.4 (2.4, 6)
Regular antimicrobial prophylactic/metaphylactic treatment at arrival	Yes	15	57.7%	3.9 ± 2.3 (3.5, 8.3)	1.2 ± 0.6 (1.5, 2.2)	2.6 ± 2 (2, 6.8)
	No	11	42.3%	1.8 ± 1.1 (1.5, 4.4)	1.3 ± 0.9 (1.1, 3)	0.5 ± 0.6 (0.1, 1.8)
Presence of a cattle crush	Yes	9	34.6%	3.5 ± 2.6 (3.5, 8.2)	1.3 ± 0.5 (1.5, 2.2)	2.2 ± 2.2 (2, 6)
	No	17	65.4%	2.7 ± 1.9 (2.6, 8.3)	1.2 ± 0.8 (1.4, 3)	1.5 ± 1.7 (0.8, 6.8)
Animal handling corridor	Yes	15	57.7%	3.2 ± 2.1 (3, 8.3)	1.3 ± 0.7 (1.5, 3)	1.9 ± 2 (1.4, 6.8)
	No	11	42.3%	2.8 ± 2.2 (2.3, 8.2)	1.3 ± 0.8 (1.4, 2.6)	1.5 ± 1.7 (0.8, 6)
Specific diet for animals in quarantine	Yes	7	26.9%	2.8 ± 2.6 (2, 8.2)	1.2 ± 0.7 (1.5, 2.2)	1.6 ± 2.1 (0.8, 6)
	No	19	73.1%	3.1 ± 2 (2.6, 8.3)	1.3 ± 0.7 (1.4, 3)	1.8 ± 1.9 (1.2, 6.8)
Fattening group divided by origin or arrival	Yes	5	19.2%	2.3 ± 1.7 (1.2, 4.4)	0.8 ± 0.6 (1, 1.5)	1.5 ± 1.6 (1, 3.9)
	No	21	80.8%	3.2 ± 2.2 (2.6, 8.3)	1.4 ± 0.7 (1.5, 3)	1.8 ± 2 (1.2, 6.8)
Fattening group divided by weight at arrival	Yes	24	92.3%	3 ± 2.2 (2.6, 8.3)	1.3 ± 0.7 (1.5, 3)	1.7 ± 2 (1, 6.8)
	No	2	7.7%	2.4 ± 2 (2.4, 3.8)	0.8 ± 1 (0.8, 1.5)	1.7 ± 0.9 (1.7, 2.3)
Daily check on animal by entering the pen	Yes	5	19.2%	3.6 ± 2.2 (2.6, 7)	1.2 ± 0.5 (1.1, 1.8)	2.4 ± 2.2 (1.9, 5.4)
	No	21	80.8%	2.9 ± 2.2 (2.3, 8.3)	1.3 ± 0.8 (1.5, 3)	1.6 ± 1.8 (1, 6.8)
All sick animals examined by veterinarian before treatment *	Yes	9	34.6%	2.1 ± 1.3 (1.5, 4.4)	1.2 ± 0.8 (1.5, 2.6)	0.9 ± 0.8 (0.8, 2.3)
	No	17	65.4%	3.5 ± 2.4 (3, 8.3)	1.3 ± 0.7 (1.4, 3)	2.1 ± 2.2 (1.9, 6.8)
Animals moved to infirmary at the first onset clinical signs	Yes	5	19.2%	2.2 ± 1.1 (1.7, 3.5)	0.8 ± 0.6 (0.8, 1.5)	1.4 ± 0.6 (1.2, 2)
	No	21	80.8%	3.2 ± 2.3 (2.6, 8.3)	1.4 ± 0.7 (1.5, 3)	1.8 ± 2.1 (0.8, 6.8)
Mechanical ventilation in closed areas	Yes	23	88.5%	3.2 ± 2.2 (3, 8.3)	1.3 ± 0.7 (1.5, 3)	1.8 ± 2 (1.4, 6.8)
	No	3	11.5%	1.7 ± 0.8 (1.3, 2.6)	0.8 ± 0.9 (0.5, 1.8)	0.9 ± 0.1 (0.8, 1)
Scraper in at least one area	Yes	13	50%	3.2 ± 2.3 (3, 8.2)	1.3 ± 0.8 (1, 3)	1.9 ± 2.1 (1.2, 6)
	No	13	50%	2.8 ± 2 (2.6, 8.3)	1.3 ± 0.7 (1.5, 2.6)	1.5 ± 1.7 (1, 6.8)
Open quarantine area	Yes	17	65.4%	2.9 ± 2.2 (2.6, 8.2)	1.2 ± 0.7 (1.1, 3)	1.7 ± 1.9 (1.2, 6)
	No	9	34.6%	3.1 ± 2.2 (2.6, 8.3)	1.3 ± 0.8 (1.5, 2.6)	1.8 ± 2 (1, 6.8)
Isolated quarantine area	Yes	23	88.5%	3 ± 2.2 (2.6, 8.3)	1.3 ± 0.7 (1.4, 3)	1.8 ± 2 (1.2, 6.8)
	No	3	11.5%	2.5 ± 1.4 (2.6, 3.8)	1.1 ± 0.9 (1.5, 1.8)	1.4 ± 0.8 (1, 2.3)
Paddock in quarantine area	Yes	6	23.1%	2.8 ± 2.4 (1.6, 7)	0.9 ± 0.4 (0.9, 1.6)	1.9 ± 2.2 (1, 5.4)
	No	20	76.9%	3 ± 2.1 (2.8, 8.3)	1.4 ± 0.7 (1.5, 3)	1.7 ± 1.8 (1.2, 6.8)
Scraper in the quarantine area	Yes	2	7.7%	2.5 ± 1.4 (2.5, 3.5)	0.9 ± 0.2 (0.9, 1)	1.6 ± 1.3 (1.6, 2.5)
	No	24	92.3%	3 ± 2.2 (2.6, 8.3)	1.3 ± 0.7 (1.5, 3)	1.7 ± 1.9 (1.1, 6.8)
Quarantine disinfection	Yes	24	92.3%	3.1 ± 2.2 (2.8, 8.3)	1.3 ± 0.7 (1.4, 3)	1.8 ± 1.9 (1.3, 6.8)
	No	2	7.7%	1.3 ± 0.9 (1.3, 2)	1.1 ± 0.6 (1.1, 1.5)	0.2 ± 0.3 (0.2, 0.4)
Open fattening area	Yes	3	11.5%	1.5 ± 0.5 (1.3, 2)	1 ± 0.5 (1, 1.6)	0.5 ± 0.4 (0.5, 0.8)
	No	23	88.5%	3.2 ± 2.2 (3, 8.3)	1.3 ± 0.7 (1.5, 3)	1.9 ± 1.9 (1.4, 6.8)
Paddock in fattening area	Yes	6	23.1%	3 ± 2.2 (2.8, 7)	1.4 ± 0.9 (1.3, 3)	1.6 ± 2.1 (0.9, 5.4)
	No	20	76.9%	3 ± 2.2 (2.5, 8.3)	1.2 ± 0.7 (1.5, 2.6)	1.8 ± 1.9 (1.1, 6.8)
Scraper in fattening area	Yes	12	46.2%	3.4 ± 2.4 (3.3, 8.2)	1.3 ± 0.8 (1.2, 3)	2.1 ± 2.1 (1.8, 6)
	No	14	53.8%	2.7 ± 2 (2.5, 8.3)	1.3 ± 0.7 (1.5, 2.6)	1.4 ± 1.7 (0.9, 6.8)
Bedding removal in fattening area	Scraper	6	23.1%	4.3 ± 2.8 (3.9, 8.2)	1.3 ± 0.7 (1.5, 2.2)	3 ± 2.2 (2.4, 6)
	Scraper and manual	6	23.1%	2.4 ± 1.4 (2.2, 4.4)	1.2 ± 0.9 (0.9, 3)	1.2 ± 1.6 (0.4, 3.9)
	Manual	14	53.8%	2.7 ± 2 (2.5, 8.3)	1.3 ± 0.7 (1.5, 2.6)	1.4 ± 1.7 (0.9, 6.8)
Fattening area disinfection	Yes	21	80.8%	3.2 ± 2.3 (2.6, 8.3)	1.3 ± 0.7 (1.4, 3)	1.9 ± 2 (1.2, 6.8)
	No	5	19.2%	2.3 ± 1.6 (2, 4)	1.2 ± 0.5 (1.5, 1.6)	1.2 ± 1.1 (0.6, 2.4)
Depopulation period in fattening area	Yes	2	7.7%	5.4 ± 4 (5.4, 8.2)	2 ± 0.3 (2, 2.2)	3.4 ± 3.7 (3.4, 6)
	No	24	92.3%	2.8 ± 1.9 (2.5, 8.3)	1.2 ± 0.7 (1.3, 3)	1.6 ± 1.7 (1.1, 6.8)
Open hospital pens	Yes	12	46.2%	2.4 ± 1.3 (2.3, 4.4)	1.1 ± 0.6 (1.3, 1.8)	1.3 ± 1.1 (1, 3.9)
	No	14	53.8%	3.5 ± 2.6 (2.8, 8.3)	1.4 ± 0.8 (1.5, 3)	2.1 ± 2.3 (1.4, 6.8)
Hospital pens isolated from rest of the barn	Yes	8	30.8%	2.4 ± 1.6 (2.2, 4.4)	1.2 ± 0.7 (1.1, 2.6)	1.2 ± 1.3 (1, 3.9)
	No	18	69.2%	3.3 ± 2.3 (2.6, 8.3)	1.3 ± 0.7 (1.5, 3)	1.9 ± 2.1 (1.1, 6.8)
Paddock in the hospital pens	Yes	1	3.8%	1.2	1.1	0.09
	No	25	96.2%	3.1 ± 2.2 (2.6, 8.3)	1.3 ± 0.7 (1.5 3)	1.8 ± 1.9 (1.2, 6.8)
Scraper in hospital pens	Yes	6	23.1%	2.5 ± 2.3 (1.6, 7)	1.2 ± 1 (0.9, 3)	1.2 ± 2.1 (0.4, 5.4)
	No	20	76.9%	3.1 ± 2.1 (2.9, 8.3)	1.3 ± 0.6 (1.5, 2.6)	1.9 ± 1.8 (1.6, 6.8)
Bedding removing in hospital pens	Scraper	3	11.5%	3.4 ± 3.1 (1.7, 7)	0.9 ± 0.6 (0.8, 1.6)	2.4 ± 2.5 (1.2, 5.4)
	Scraper and manual	3	11.5%	1.6 ± 1.3 (1.1, 3)	1.6 ± 1.3 (1, 3)	0.03 ± 0.05 (0, 0.08)
	Manual	20	77%	3.1 ± 2.1 (2.9, 8.3)	1.3 ± 0.6 (1.5, 2.6)	1.9 ± 1.8 (1.6, 6.8)
Hospital pens disinfection	Yes	24	92.3%	3.1 ± 2.2 (2.8, 8.3)	1. ± 0.7 (1.5, 3)	1.8 ± 1.9 (1.2, 6.8)
	No	2	7.7%	1.2 ± 0.7 (1.2, 1.7)	0.6 ± 0.1 (0.6, 0.7)	0.6 ± 0.9 (0.6, 1.2)
Depopulation period in hospital pens	Yes	10	38.5%	3.2 ± 2 (3.1, 8.2)	1.3 ± 0.6 (1.5, 2.2)	2 ± 1.8 (1.6, 6)
	No	16	61.5%	2.8 ± 2.2 (2.2, 8.3)	1.3 ± 0.8 (1.3, 3)	1.6 ± 2 (0.8, 6.8)

* Farms in the category “No” did not necessarily treat all animals without a previous clinical examination by a veterinarian, but this possibility was discussed with the practitioner following the farm, who left specific treatment plans for the farmer.

**Table 3:** Consumption of active substance (kg) by drug group and route of administration (oral and parenteral) from 26 fattening operations located in northwestern Italy, for the years 2014 and 2015. In bold are the highest priority critically important antimicrobials (HPCIA) in category B according to the European Medicine Agency [26].

Substance Group	Parenteral			Oral			Total		
	No. Farms	kg	%	No. Farms	kg	%	No. Farms	kg	%
Aminoglycosides	22	20.1	5.7	0	0	0	22	20.1	2.4
Penicillines	22	22.9	6.5	0	0	0	22	22.9	2.8
**3rd and 4th generation Cephalosporines**	**12**	**3.1**	**0.9**	**0**	**0**	**0**	**12**	**3.1**	**0.4**
Fenicoles	24	136.1	38.7	0	0	0	24	136.1	16.5
**Fluoroquinolones**	**25**	**23.5**	**6.7**	**1**	**0.04**	**0.01**	**25**	**23.5**	**2.9**
Lincosamides	18	8.4	2.4	0	0	0	18	8.4	1
Macrolides	26	83.7	23.8	0	0	0	26	83.7	10.2
**Polymyxins**	**3**	**0.2**	**0.1**	**0**	**0**	**0**	**3**	**0.2**	**0.1**
Sulfonamides	15	14.8	4.2	0	0	0	15	14.8	1.8
Sulfonamide and Trimethoprim	15	18.4	5.2	5	40.3	8.6	17	58.7	7.1
Tetracyclines	20	20.4	5.8	17	429.7	91.4	21	450.2	54.8
Total	26	351.6	42.8	17	470.1	57.2	-	821.7	100

**Table 4:** Distribution, mean ± SD, median and maximum nADDa administered per year in 2014 and 2015. Data from 26 fattening operations located in northwestern Italy are reported by active substance group and individual treatments and route of administration (oral, parenteral), and number of farms, The long-acting (LA) factors and the final ADD (mg/kg) used for the analysis are also reported. Percentage of oral and parenteral formulation refers to the type of treatment (“group” vs. “individual”), while percentage of active compounds refers to the type of formulation (“oral” vs. ”parenteral”). In bold are the highest priority critically important antimicrobials (HPCIA) in category B according to the European Medicine Agency [26].

					nADDa			
Active Substance	ATC-Vet	LA Factor	ADD	No. of Farms	%	Mean ± SD	Median	Max
**GROUP TREATMENTS**					57.5			
*Oral formulation*					70.5			
Doxycycline	QJ01AA02	1	10	17	97	1.17 ± 1.7	0.3	5.6
Sulfadiazine/trimethoprim	QJ01EW10	1	24	5	3	0.04 ± 0.1	0	0.4
*Parenteral formulation*					29.5			
Aminosidin	QJ01GB92	1	10.5	1	0.8	0.004 ± 0.02	0	0.1
Amoxicillin	QJ01CA04	1–2	7.25	1	0.08	0.0004 ± 0.002	0	0.01
**Enrofloxacin**	**QJ01MA90**	**1**–**2**	**4.4**	**3**	**3.2**	**0.02 ± 0.07**	**0**	**0.4**
Florfenicol-Florfenicol + flunixin	QJ01BA90/ QI01BA99	2–4	12.5	13	6.9	0.04 ± 0.08	0.004	0.4
Lincomycin/spectinomycin	QJ01FF52	1	15	4	0.2	0.001 ± 0.005	0	0.02
**Marbofloxacin**	**QJ01MA93**	**1**–**4**	**2**	**8**	**6.2**	**0.03 ± 0.1**	**0**	**0.5**
Oxytetracycline	QJ01AA06	1–2	6.5	7	4	0.02 ± 0.09	0	0.4
Procaine benzylpenicillin	QJ01CE09	1	12	1	0.04	0.0002 ± 0.001	0	0.006
Spiramycin	QJ01FA02	2	15.6	5	1.7	0.009 ± 0.02	0	0.08
Sulfadimidine/Trimethoprim	QJ01EW03	1	15.5	1	0.2	0.001 ± 0.005	0	0.03
Sulfamonomethoxine	QJ01EQ18	1	40	1	0.06	0.0003 ± 0.002	0	0.008
Thiamphenicol	QJ01BA02	1	37.5	1	0.03	0.0002 ± 0.0008	0	0.004
Tildipirosin	QJ01FA96	5	0.8	13	26.8	0.14 ± 0.4	0.006	1.6
Tilmicosin	QJ01FA91	1–2	6	6	5.9	0.03 ± 0.1	0	0.5
Tylosin	QJ01FA90	1	7	4	2.5	0.01 ± 0.04	0	0.2
Tulathromycin	QJ01FA94	5	0.5	12	41.4	0.2 ± 0.4	0	1.8
**INDIVIDUAL TREATMENTS**					42.5			
*Oral formulation*					1.9			
Doxyciclyne	QJ01AA02	1	10	6	98.8	0.02 ± 0.09	0	0.4
**Enrofloxacin**	**QJ01MA90**	**1**	**3.75**	**1**	**1.2**	**0.0003 ± 0.002**	**0**	**0.008**
*Parenteral formulation*					98.1			
Aminosidin	QJ01GB92	1	10.5	2	0.2	0.003 ± 0.01	0	0.05
Amoxicillin	QJ01CA04	1–2	7.25	13	3.9	0.05 ± 0.07	0.02	0.3
Ampicillin	QJ01CA01	1	7.5	6	1.4	0.02 ± 0.05	0	0.2
Ampicillin**/** **colistin sulphate**	**QJ01RV01**	**1**	**11.3**	**3**	**0.3**	**0.003 ± 0.01**	**0**	**0.04**
Ampicillin/dicloxacillin	QJ01CR50	1	10.7	7	0.9	0.01 ± 0.03	0	0.2
Procaine benzylpenicillin/dihydrostreptomycin	QJ01RA01	1	43	3	0.1	0.001 ± 0.005	0	0.02
**Cefquinome**	**QJ01DE90**	**1**	**1**	**4**	**1.2**	**0.02 ± 0.06**	**0**	**0.3**
**Ceftiofur**	**QJ01DD90**	**1**–**6**	**1**	**9**	**6.3**	**0.08 ± 0.3**	**0**	**1.2**
**Danofloxacin**	**QJ01MA92**	**2**	**3**	**2**	**0.07**	**0.0009 ± 0.004**	**0**	**0.02**
**Enrofloxacin**	**QJ01MA90**	**1**–**2**	**4.4**	**11**	**6.4**	**0.08 ± 0.2**	**0**	**0.8**
Erythromycin/sulfamonomethoxine	QJ01RA91	1	25	1	0.05	0.0007 ± 0.003	0	0.02
Florfenicol-Florfenicol + flunixin	QJ01BA90/QI01BA99	2–4	12.5	22	19.9	0.25 ± 0.2	0.2	0.6
Gamithromycin	QJ01FA95	5	1.2	1	0.01	0.0001 ± 0.0006	0	0.003
Kanamycin	QJ01GB04	0.5	13.5	5	0.6	0.008 ± 0.03	0	0,2
Lincomycin/spectinomycin	QJ01FF52	1	15	18	2.9	0.04 ± 0.05	0.01	0.2
**Marbofloxacin**	**QJ01MA93**	**1**–**4**	**2**	**18**	**19.5**	**0.2 ± 0.3**	**0.1**	**1**
Oxytetracycline	QJ01AA06	1–2	6.5	20	6.3	0.08 ± 0.08	0.07	0.3
Procaine benzylpenicillin	QJ01CE09	1	12	1	0.06	0.0008 ± 0.004	0	0.02
Spiramycin	QJ01FA02	2	15.6	16	6.7	0.08 ± 0.1	0.05	0.4
Sulfadiazine/trimethoprim	QJ01EW10	1	24	1	0.1	0.001 ± 0.007	0	0.04
Sulfadimethoxine	QJ01EQ09	1	31	4	0.3	0.003 ± 0.01	0	0.05
Sulfadimidine/sulfadimethoxine/trimethoprim	QJ01EW03	1	15.5	1	0.02	0.0003 ± 0.001	0	0.007
Sulfadimidine/trimethoprim	QJ01EW03	1	15.5	15	2.3	0.03 ± 0.04	0.01	0.1
Sulfametopyrazine	QJ01EQ19	1	36	1	0.3	0.003 ± 0.02	0	0.08
Sulfamonomethoxine	QJ01EQ18	1	40	10	0.6	0.008 ± 0.02	0	0.06
Thiamphenicol	QJ01BA02	1	37.5	8	0.5	0.006 ± 0.02	0	0.06
Tildipirosin	QJ01FA96	5	0.8	12	5.3	0.07 ± 0.09	0	0.4
Tilmicosin	QJ01FA91	1–2	6	15	0.8	0.01 ± 0.02	0.004	0.06
Tylosin	QJ01FA90	1	7	23	12.4	0.15 ± 0.2	0.1	0.8
Tulathromycin	QJ01FA94	5	0.5	6	0.7	0.009 ± 0.03	0	0.1

**Table 5:** Dose (mg/kg) recommended by pharmaceutical manufacturer and dosing ratio of individual and group antimicrobial treatments by route of administration, in 26 Piedmontese fattening operations. More than one recommended dose was reported when more than one commercial formulation of the same active substance was present (e.g., long-acting and normal preparations). RD denotes recommended dose; UDD denotes used daily dose. In bold are the highest priority critically important antimicrobials (HPCIA) in category B according to the European Medicine Agency [26].

Active Substance	ATC-Vet	RD	Mean ± SD(Min, Max)		No. of Farms		
			UDD	UDD/RD	<0.8	0.8–1.2	>1.2
**GROUP TREATMENTS**							
*Oral formulation*							
Doxycycline	QJ01AA02	10	12.5 ± 5.7 (5.8, 29.3)	1.2 ± 0.6 (0.6, 2.9)	2	9	6
Sulfadiazine/Trimethoprim	QJ01EW10	24	12.8 ± 3.6 (7.7, 17.3)	0.5 ± 0.2 (0.3, 0.7)	5	-	-
*Parenteral formulation*							
Aminosidin	QJ01GB92	10.5	1.8	0.2	1	-	-
Amoxicillin	QJ01CA04	15	0.6	0.04	1	-	-
**Enrofloxacin**	**QJ01MA90**	**5**–**7.5**	**1.5 ± 0.5 (1.2, 2.1)**	**0.3 ± 0.1 (0.2, 0.4)**	**3**	**-**	**-**
Florfenicol/Florfenicol + flunixin	QJ01BA90/QI01BA99	20–40	11.6 ± 5.1 (3.1, 19.3)	0.5 ± 0.2 (0.2, 1)	12	1	-
Lincomycin/Spectinomycin	QJ01FF52	15	6.1 ± 2.3 (3.5, 9)	0.4 ± 0.2 (0.2, 0.6)	4	-	-
**Marbofloxacin**	**QJ01MA93**	**2**–**8**	**4.5 ± 3.4 (1, 11)**	**1.2 ± 1.3 (0.1, 3.8)**	**4**	**1**	**3**
Oxytetracycline	QJ01AA06	4.7–7–20	3.1 ± 2.9 (0.3, 7.1)	0.4 ± 0.5 (0.06, 1.4)	6	-	1
Procaine benzylpenicillin	QJ01CE09	12	12	1	-	1	-
Spiramycin	QJ01FA02	31.25	7.2 ± 4.8 (1.7, 13.7)	0.2 ± 0.2 (0.05, 0.4)	5	-	-
Sulfamidine/Trimethoprim	QJ01EW03	15	11	0.7	1	-	-
Sulfamonomethoxine	QJ01EQ18	40	2.5	0.06	1	-	-
Thiamphenicol	QJ01BA02	37.5	12.5	0.3	1	-	-
Tildipirosin	QJ01FA96	4	2.8 ± 1 (1, 4.3)	0.7 ± 0.3 (0.3, 1.1)	8	5	-
Tilmicosin	QJ01FA91	10	6.7 ± 2.1 (4.5, 10.4)	0.7 ± 0.2 (0.5, 1)	5	1	-
Tylosin	QJ01FA90	7	7.7 ± 6.4 (2, 14.5)	1.1 ± 0.9 (0.3, 2.1)	2	-	2
Tulathromycin	QJ01FA94	2.5	1.8 ± 0.4 (1.2, 2.5)	0.7 ± 0.2 (0.5, 1)	8	4	-
**INDIVIDUAL TREATMENTS**							
*Oral formulation*							
Doxyciclyne	QJ01AA02	10	22 ± 15.4 (9.4, 47)	2.2 ± 1.5 (0.9, 4.7)	-	-	4
**Enrofloxacin**	**QJ01MA90**	**3.75**	**1.9**	**0.5**	**1**	**-**	**-**
*Parenteral formulation*							
Aminosidin	QJ01GB92	10.5	8.6 ± 2.3 (7, 10.2)	0.8 ± 0.2 (0.7, 1)	1	1	-
Amoxicillin	QJ01CA04	7–15	9.7 ± 3.3 (6.6, 18.3)	1.1 ± 0.6 (0.4, 2.6)	5	5	3
Ampicillin	QJ01CA01	7.5	13.2 ± 5.3 (5, 21.4)	1.8 ± 0.7 (0.7, 2.9)	1	-	5
Ampicillin/**colistin sulphate**	**QJ01RV01**	**11.2**	**6.5 ± 1.5 (4.8, 7.3)**	**0.6 ± 0.1 (0.4, 0.7)**	**3**	**-**	**-**
Ampicillin/dicloxacillin	QJ01CR50	10.7	10.4 ± 3.1 (6.9, 15.6)	1 ± 0.3 (0.6, 1.5)	2	3	2
Procaine benzylpenicillin/Dihydrostreptomycin	QJ01RA01	19.5–40	20.1 ± 2.4 (18.3, 22.2)	0.6 ± 0.4 (0.5, 1.3)	2	1	-
**Cefquinome**	**QJ01DE90**	**1**	**1 ± 0.3 (0.5, 1.3)**	**1 ± 0.3 (0.5, 1.3)**	**1**	**2**	**2**
**Ceftiofur**	**QJ01DD90**	**1**–**6.6**	**3.5 ± 2.6 (0.8, 7.8)**	**1.7 ± 2.1 (0.5, 7.2)**	**3**	**4**	**2**
**Danofloxacin**	**QJ01MA92**	**6**	**6.2 ± 0.7 (5.7, 6.7)**	**1 ± 0.1 (1, 1.1)**	**-**	**2**	**-**
**Enrofloxacin**	**QJ01MA90**	**5**–**7.5**	**3.1 ± 1 (1.4, 5.2)**	**0.6 ± 0.2 (0.2, 1)**	**10**	**1**	**-**
Erythromycin/Sulfamonomethoxine	QJ01RA91	25	13.1	0.5	1	-	-
Florfenicol/Florfenicol + flunixin	QJ01BA90/QI01BA99	20–30–40	15.6 ± 4 (8.1, 22.2)	0.7 ± 0.2 (0.4, 1.1)	16	4	-
Gamithromycin	QJ01FA95	6	3.5	0.6	1	-	-
Kanamycin	QJ01GB04	16.5	6.2 ± 1.9 (4, 8.6)	0.4 ± 0.1 (0.3, 0.5)	2	2	1
Lincomycin/spectinomycin	QJ01FF52	15	9.6 ± 2.5 (5.6, 13.6)	0.6 ± 0.2 (0.4, 0.9)	14	4	-
**Marbofloxacin**	**QJ01MA93**	**2**–**8**	**4.8 ± 2.1 (1.6, 10.2)**	**1.1 ± 0.9 (0.2, 3.1)**	**7**	**5**	**6**
Oxytetracycline	QJ01AA06	4.7–7–20	7.3 ± 2.3 (3.9, 13.5)	1.3 ± 0.5 (0.6, 2.3)	6	4	11
Procaine benzylpenicillin	QJ01CE09	12	12.4	1	-	1	-
Spiramycin	QJ01FA02	31.25	11.2 ± 2.1 (7.8, 15.3)	0.4 ± 0.07 (0.3, 0.5)	16	-	-
Sulfadiazine/trimethoprim	QJ01EW10	20	29.9	1.5	-	-	1
Sulfadimethoxine	QJ01EQ09	31	29.8 ± 21.2 (4.4, 56.3)	1 ± 0.6 (0.1, 1.8)	1	2	1
Sulfamidine/ sulfadimethoxine/trimethoprim	QJ01EW03	14.4	11.7	0.8	-	1	-
Sulfadimidine/trimethoprim	QJ01EW03	15–16	17.9 ± 3.7 (11.6, 24.3)	1.2 ± 0.3 (0.8, 1.6)	1	5	9
Sulfametopyrazine	QJ01EQ19	36	21.4	0.6	1	-	-
Sulfamonomethoxine	QJ01EQ18	40	33.4 ± 8.6 (23.5, 52.1)	0.8 ± 0.2 (0.6, 1.3)	6	3	1
Thiamphenicol	QJ01BA02	37.5	14.2 ± 2.8 (9.3, 17.4)	0.4 ± 0.08 (0.3, 0.5)	8	-	-
Tildipirosin	QJ01FA96	4	4.1 ± 0.9 (2.9, 5.8)	1 ± 0.2 (0.7, 1.5)	1	9	2
Tilmicosin	QJ01FA91	7–10	7.2 ± 3.5 (3, 15.2)	0.7 ± 0.4 (0.3, 1.5)	10	3	2
Tylosin	QJ01FA90	7	11.6 ± 2.4 (7.4, 15.9)	1.7 ± 0.4 (1.1, 2.3)	-	9	14
Tulathromycin	QJ01FA94	2.5	2.2 ± 0.4 (1.5, 2.7)	0.9 ± 0.2 (0.6, 1.1)	1	5	-

## Data Availability

Data available upon request to the corresponding author.
